# Early Behavioral Abnormalities and Perinatal Alterations of PTEN/AKT Pathway in Valproic Acid Autism Model Mice

**DOI:** 10.1371/journal.pone.0153298

**Published:** 2016-04-12

**Authors:** Eun-Jeong Yang, Sangzin Ahn, Kihwan Lee, Usman Mahmood, Hye-Sun Kim

**Affiliations:** 1 Department of Pharmacology and Biomedical Sciences, Seoul National University College of Medicine, Seoul, Republic of Korea; 2 Department of Pharmacology, Inje Univeirsity College of Medicine, Busan, Republic of Korea; 3 Interdisciplinary Program in Brain Sciences, Seoul National University College of Natural Sciences, Seoul, Republic of Korea; 4 Seoul National University Bundang Hospital, Seoul National University College of Medicine, Sungnam, Republic of Korea; The George Washington University, UNITED STATES

## Abstract

Exposure to valproic acid (VPA) during pregnancy has been linked with increased incidence of autism, and has repeatedly been demonstrated as a useful autism mouse model. We examined the early behavioral and anatomical changes as well as molecular changes in mice prenatally exposed to VPA (VPA mice). In this study, we first showed that VPA mice showed developmental delays as assessed with self-righting, eye opening tests and impaired social recognition. In addition, we provide the first evidence that primary cultured neurons from VPA-treated embryos present an increase in dendritic spines, compared with those from control mice. Mutations in phosphatase and tensin homolog (PTEN) gene are also known to be associated with autism, and mice with PTEN knockout show autistic characteristics. Protein expression of PTEN was decreased and the ratio of p-AKT/AKT was increased in the cerebral cortex and the hippocampus, and a distinctive anatomical change in the CA1 region of the hippocampus was observed. Taken together, our study suggests that prenatal exposure to VPA induces developmental delays and neuroanatomical changes via the reduction of PTEN level and these changes were detectable in the early days of life.

## Introduction

Autism spectrum disorder (ASD) is a group of developmental disabilities characterized by social interaction, verbal and nonverbal communication, and stereotyped behaviors and interests [1]. Its prevalence is as high as 0.7–1.1% in the general population and is four times more common in males than females [2–4]. Abnormal development is often observed in autistic patients in the early stages of life, including weight fluctuation [5, 6], abnormal brain development [7–9], disruption in synaptic connection and hyperactive neuronal connections resulting in behavioral complexities [10–12]. While up to 25% of ASD cases are identified to carry inheritable single genes or rare gene mutations [13–16], population studies suggest that environmental factors during the prenatal period also contribute to an increased incidence of autism [4, 17, 18].

Valproic acid (VPA), an antiepileptic agent used to treat epilepsy and bipolar disorder, is also associated with an increased risk for congenital malformations and delayed cognitive development in offsprings [19–21]. Prospective and retrospective studies have demonstrated that the exposure to VPA during pregnancy is associated with a three-fold rate of major anomalies and dysmorphic features as well as decreased intrauterine growth [22]. Epidemiological data has been successfully implanted into research as animal studies using male VPA-exposed mice have shown repeatedly core behavioral signs of autism as well as molecular changes linked to the disorder [23–26]. The underlying molecular mechanisms of VPA-treated mice have been explored to imply autism-related genes including brain-derived neurotrophic factor [26], neuroligin 1 [27], neuroligin 3 [28, 29], and monoamine synaptic transmission [30, 31].

Phosphatase and tensin homolog (PTEN), a gene located on chromosome 10q23, is involved in a wide variety of cellular processes relevant to brain growth and circuit function [32, 33]. PTEN, previously recognized as a tumor suppressor gene mutated in many human cancers [34], has recently gained traction in its association with ASD [32, 35–39]. PTEN mutation was recently documented as a causative factor and its conditional knockout studies are validating the link between autism and PTEN [32, 37, 38]. *Pten* gene is considered as susceptible for autism as Fragile X protein (FXS) and Tuberous sclerosis protein complex 1 and 2 (TSC1/2 complex), and PTEN mutations may account as much as 5% of autism associated with macrocephaly and 1% of autism [40]. Perturbation in downstream pathway of PTEN, the phosphoinositide 3-kinase (PI3K)/protein kinase B (AKT)/mechanistic target of rapamycin (mTOR) pathway, results in behavioral abnormalities and is expected to play a significant role in ASD [35, 37, 38, 41]. In order to gain insight in an environmental inducer of autism, we explored the possibility of VPA’s in utero exposure in relations to PTEN expression.

Although ASD is generally considered to be a developmental disorder, behavioral alteration in the early postnatal phase have yet to be extensively studied in the VPA-induced autism model. In this study, we focused on the early behavioral, anatomical, and molecular changes similar to those found in previously reported PTEN conditional knockout mice [37, 38]. In addition, we analyzed the changes in dendritic spine density by employing primary neuronal cultures from VPA-exposed mice.

## Materials and Methods

### Experimental animals

Fourteen pregnant BALB/c (Central Lab Animal Inc., Korea) pregnant mice were randomly assigned to VPA-injected (VPA group, *n* = 9) or saline-injected (SAL group, *n* = 5) groups. The VPA group received a single subcutaneous injection of 600 mg/kg valproic acid sodium salt (Sigma, MO, USA) on embryonic day 13 (E13) [42, 43], while the control group (SAL) received an equal amount of saline injection. Two SAL pregnant mice and 4 VPA pregnant mice were sacrificed on E18 to obtain brain sample of E18 fetuses. The remaining pregnant mice were housed in individual cages and left undisturbed until postnatal day 5 (P5) to prevent cannibalism. Among 51 female and male pups, only 21 male mice (SAL, n = 10; VPA, n = 11) were used in this study. All animal procedures were performed following the National Institutes of Health Guidelines for the Humane Treatment of Animals, with approval from the Institutional Animal Care and Use Committee of Seoul National University (IACUC approval number SNU-130319-01).

### Behavioral tests

BALB/c mice were housed on a 12 h light/dark cycle in a temperature-controlled environment. Taking into consideration that some VPA-induced behavioral changes are more apparent in males [24] and that human ASD is more prevalent in males, male mice were subjected to behavioral studies.

#### 1) Self-righting test

Self-righting test was held on P5-9 as described in previous literature [42]. Each mouse was placed on its back and gently held with all four limbs extended outwards at which time it was released. Time to right was recorded by the latency for all four paws touching the surface. A maximum score of 30 s was recorded when the mouse failed to right in that period. The test was performed by an investigator blinded to the groups.

#### 2) Eye opening test

From P12 to P16, mice were inspected daily for eye opening [26]. Pups were inspected daily if the eyes were opened. 1 point was scored for each eye, resulting with a score from 0 to 2 for each pup.

#### 3) Maternal scent preference test

Maternal scent preference test was conducted on P14 as described in previous research with minor modifications [44]. Each pup was moved from home litter to a transparent polycarbonate cage (20 × 30 × 15 cm). The left third of the test cage was filled to a depth of 3 cm with litter from the mother’s cage, the center third contained clean litter, and the right third contained litter from the cage of a stranger dam. The position of the test litters (mother and stranger) was alternated across subjects to control for any side preferences. Three 1 min trials, with inter-trial intervals of 10 sec, were administered for each pup. For the first trial, pups were placed in the center of the fresh litter facing the back wall of the test cage. For the second trial, pups were placed in the center of the fresh litter facing the section containing its mother’s cage litter. For the third trial, pups faced the section containing the litter of the stranger dam. Time spent in each section of the cage was recorded and averaged across the three trials. The pup was considered to be inside a section when all four paws were touching the litter within the specified region. Video analysis of maternal scent preference test was performed by an investigator blinded to the groups.

### Tissue preparation

Tissue preparation was performed as previously described [45]. Briefly, animals were anesthetized and immediately cardiac-perfused with heparinized phosphate buffered saline (PBS). One hemisphere was fixed in 4%-paraformaldehyde solution and was sectioned for histological studies, while the other hemisphere was lysed in RIPA buffer with a cocktail of protease inhibitors (Complete Protease Inhibitor, Roche, Switzerland).

### Primary neuron cultures

Six pregnant C57BL/6N mice (Koatech, Korea) were randomly assigned to VPA (*n* = 3) or SAL (*n* = 3) groups, and were injected with 600mg/kg valproic acid sodium salt solution or an identical volume of saline on E13. Mouse primary neuron cultures were prepared on E18 by dissecting the hippocampus and cerebral cortex from the fetal brains, followed by dissociation with 0.25% trypsin and plating onto 18 mm coverslips coated with poly-L-lysine. During dissection, a piece of the cerebellum was obtained for sexing by PCR [46]. The neurons from male fetuses were cultured in Neurobasal medium supplemented with B27, 2 mM GlutaMAX-I supplement and 100 μg/ml penicillin/streptomycin (all reagents obtained from Invitrogen) at 37°C in a humidified environment of 95% air/5% CO_2_. The body and brain weight of fetuses were documented before performing dissection.

### Dendritic spine density analysis

Dendritic spine density analysis was performed as previously described [47]. Briefly, primary cortical neuron cultures (*days in vitro* 12–13, DIV 12–13) were transfected with mCAG-IRES-mGFP vector for visualization. The number of dendritic spines was evaluated at DIV 19–20. The fluorescent images were acquired with an LSM 510 confocal microscope (Carl Zeiss, Germany), and the settings were kept consistent for all samples. The dendritic spines were counted on segments of secondary dendrites which are 50–100 μm apart from the center of the cell soma by an investigator blinded to the groups.

### Western blotting

For western blotting, brain samples were lysed by RIPA buffer and loaded onto 8% SDS-PAGE gels and transferred to nitrocellulose membranes (Millipore, MA, USA). Membranes were then incubated in 5% bovine serum albumin for 1 h at room temperature followed by overnight incubation with appropriate primary antibodies (PTEN antibody, sc-7974, Santa Cruz Biotechnology, CA, USA; phosphorylated-AKT (p-AKT) antibody, #9271, Cell Signaling Technology, MA, USA; AKT antibody, sc-8312, Santa Cruz Biotechnology; beta-actin antibody, sc-47778, Santa Cruz Biotechnology). The membranes were then incubated for 1 h at room temperature with secondary antibodies conjugated with HRP (Invitrogen, CA, USA). The HRP signals were visualized by WestSave chemiluminescent detection kit (AbFrontier, South Korea) and went through densitometric analysis on ImageJ software.

### Histological studies

For histological analysis, 20 μm-thick coronal sections containing the cortex and hippocampus were obtained using a cryostat (Thermo Scientific, MA, USA) and mounted on slides. For Nissl staining, sections were stained with 0.1% cresyl violet (Sigma, MO, USA) solution for 10 min, then rinsed quickly in distilled water and differentiated in 95% ethanol for 20 min. For hematoxylin and eosin (H&E) staining, the sections were stained with hematoxylin (Sigma, MO, USA) and washed in tap water. Then the sections were placed in 1% HCl solution with 80% ethanol, washed again in tap water, and then stained with eosin for 10 min. After additional washing and dehydration steps, the slides were mounted and protected with a coverslip. Digitized images of Nissl and H&E stain were obtained by an optical microscope using the same settings for all samples.

To obtain immunofluorescence data, sections (20 μm) containing cortex and hippocampus were obtained by a cryostat (Shandon Cryotome FE, Thermo Scientific, MA, USA), and mounted on slides. The slides were boiled in pH 8.5 citric acid for 1 h and then blocked in a blocking solution containing 5% horse serum, 5% bovine serum albumin, and 0.03% triton X-100. Sections were then incubated with the following antibody and ratio, 1:200 PTEN, 1:200 MAP2, and 1:10,000 4',6-diamidino-2-phenylindole (DAPI). After the overnight incubation, samples were washed 3 times with 1x PBS and were incubated in secondary antibodies for 1 hr at room temperature. The fluorescence signals were visualized with a confocal microscope (LSM510, Carl Zeiss, Germany) using the same settings for all samples.

### Statistical analysis

Data were expressed as mean ± standard error of the mean (SEM). Statistical analysis was performed by two-way repeated measures ANOVA and two-tailed Student’s *t*-test on SPSS 22 software (IBM, NY, USA). The difference among groups was considered significant for *, *p*<0.05, **, *p*<0.01, and ***, *p*<0.001.

## Results

### VPA mice showed developmental delays

**Fig 1:**
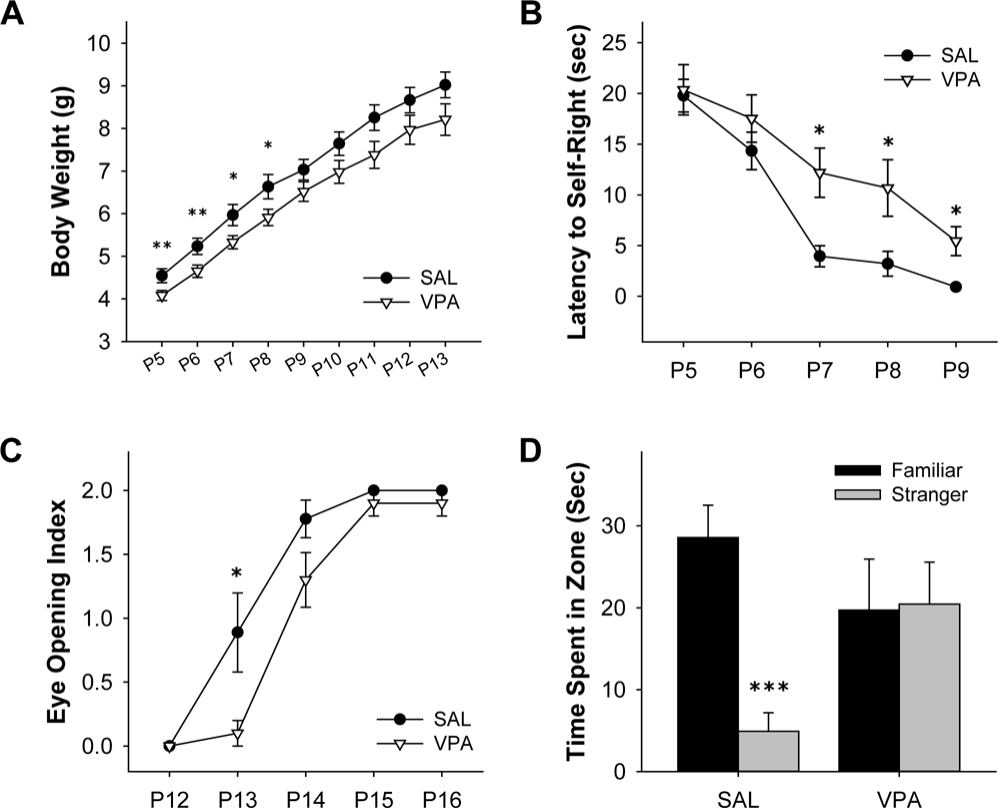
VPA mice showed developmental delays and social recognition impairments. Body weight, self-righting reflex, eye opening was checked to determine if VPA mice show behavioral developmental delays, while maternal scent preference was conducted to confirm social recognition impairments. **(A)** VPA mice showed significantly decreased body weight from on P5-9. **(B)** VPA mice showed increased latency to self-right on P7-9. **(C)** VPA mice showed lower eye opening index scores on P13. **(D)** SAL mice spent more time in the zone with familiar scent, while VPA mice did not show difference in time spent in each zone. (**A-C**, * significantly different between SAL and VPA mice on same date, * *p* < 0.05, ** *p* < 0.01. **D**, * significantly different between zones, *** *p* < 0.001. *n* = 9 for SAL, *n* = 10 for VPA in all experiments.)

VPA increases the risk of teratogenic effects characterized by minor malformations and developmental delays [48, 49]. To gain insight in the ASD developmental profile and its effects we documented several developmental milestones with VPA-exposed mice (VPA mice). Physical malformations, such as tail kinks, are reported to be observed in VPA mice [50], but the pups did not display any visible deformities. Early detection of ASD has gained significance and early developmental markers, such as low body weight, have been shown to correlate with ASD [51]. Body weight of SAL and VPA mice have been checked daily from P5 to P13. Two-way repeated measures ANOVA indicated a statistical significance for date (*p* < 0.001 with *F*(1.70, 28.88) = 488.70) as well as for group (*p* < 0.05 with *F*(1, 17) = 4.48). There was no significant interaction between date and group (*p* = 0.50 with *F*(1.70, 28.88) = 0.67). By applying separate *t*-tests on each date, we found that VPA pups showed significantly decreased body weight from P5 to P8 (P5, *t*(17) = 3.28, *p* < 0.01; P6, *t*(17) = 2.99, *p* < 0.01; P7, *t*(14.23) = 2.41, *p* < 0.05; P8, *t*(14.63) = 2.23, *p* < 0.05; *n* = 9, 10) (Fig 1A).

In order to determine if VPA mice showed behavioral developmental delays in early age, we measured the duration of self-righting reflex on P5-9. Two-way repeated measures ANOVA showed a statistical significance for both date (*F*(4, 68) = 26.31, *p* < 0.001) and group (*F*(1, 17) = 7.77, *p* < 0.05). There was no significant interaction between date and group (*p* = 0.31 with *F*(4,68) = 1.21). While the time to self-right decreased as days passed, separate *t*-tests resulted in significant delay of time to self-right in VPA mice on P7-9 (P7, *t*(17) = -2.37, *p* < 0.05; P8, *t*(17) = -2.36, *p* < 0.05; P9, t(9.07) = -3.17, *p* < 0.05; *n* = 9, 10) (Fig 1B). Eye opening has been documented to occur on P12-16 and is a well-studied developmental milestone, which represents neurodevelopment in early days of life. Two-way repeated measures ANOVA indicated a statistical significance for date (*F*(2.40, 40.73) = 105.23, *p* < 0.001) and group (*F*(1, 17) = 6.28, *p* < 0.05). There was also a significant interaction between date and group (*F*(2.40, 40.73) = 3.65, *p* < 0.05). Further analyzing each date with separate *t*-tests, both groups showed no difference at the beginning and completion of eye opening, while VPA mice showed significant delay on P13 (*t*(9.67) = 2.43, *p* < 0.05; *n* = 9, 10) (Fig 1C) which concurs with previous studies [23, 28]. These data provide further evidence of early developmental delays observed in VPA exposed pups.

### VPA mice showed social recognition impairments

Impairment in social interaction is one of the core symptoms in ASD, and three-chamber assay is one of the most common tests in determining social interaction [52, 53]. However, since the three-chamber paradigm is applied to juvenile or adult mice, we used a modified version of maternal scent preference test in order to determine early behavioral impairments in social recognition. SAL pups spent more time on familiar bedding (*t*(16) = 5.20, *p* < 0.001, *n =* 9 per group), while VPA pups showed no statistical difference in time spent on familiar or stranger zones (*t*(16) = -0.06, *p* = 0.957, *n =* 10 per group) (Fig 1D). This data suggests that VPA pups are incapable of distinguishing between familiar and stranger scents, and thus reiterating the dysfunctional social recognition commonly seen in ASD.

### Neuroanatomical abnormalities were observed in VPA mice

**Fig 2:**
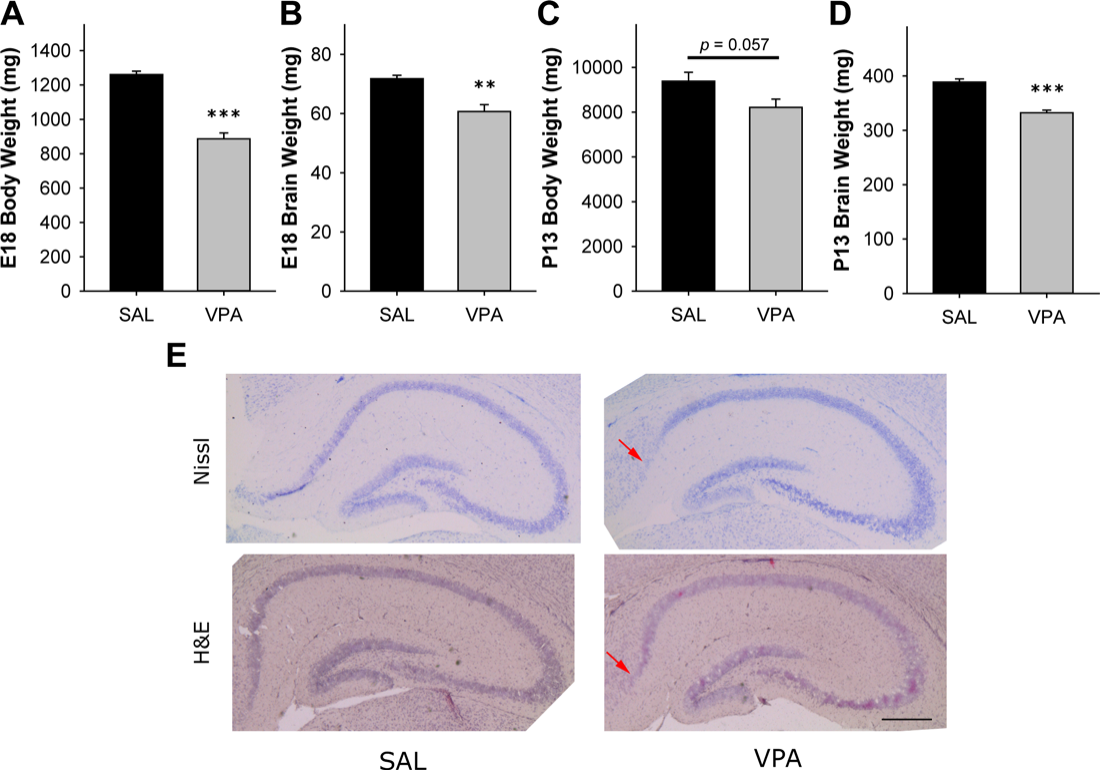
VPA mice showed physical and anatomical changes. **(A-D)** Body and brain weight was decreased in VPA mice on E18 and P13. **(E)** Representative pictomicrographs of Nissl staining and H&E staining of the hippocampus. Arrows indicate hypocellularity in the CA1-subiculum region of the hippocampus. Scale bar indicates 500 μm. (**A-D** * Significantly different among groups, ** *p* < 0.01, *** *p* < 0.001, *n* = 9 for SAL, n = 10 VPA for behavioral tests.)

Multiple studies suggest that macrocephaly is a viable marker in detecting ASD and mental retardation [9, 54]. Paradoxically, some studies show correlation between reduced brain weight and ASD [55, 56]. To identify the effects of *in utero* exposure to VPA on brain development, the brains and bodies of embryonic day 18 (E18) fetuses as well as pups on P13 were weighed. VPA-exposed fetuses had a significantly reduced brain weight compared to SAL at E18 (*t*(11.67) = 4.27, *p* < 0.001, *n* = 10, 9) (Fig 2A) as well as body weight (*t*(17) = 9.73, *p* < 0.01, *n* = 10, 9) (Fig 2B). Although the brain and body weight was decreased in E18 VPA fetuses, the brain weight to body weight ratio was paradoxically increased (SAL = 0.0571 ± 0.0014, VPA = 0.0687 ± 0.0023, *t*(17) = -4.52, *p* < 0.001, *n* = 10, 9). Interestingly, the discrepancy in brain weight between SAL and VPA was also persistent on P13 (*t*(13) = 7.04, *p* < 0.001, *n* = 5, 10) (Fig 2D). However, difference in body weight did not reach significance on P13 (*t*(10.31) = 2.14, *p* = 0.057, *n* = 5, 10) (Fig 2C).

The limbic system is known to mediate memory and social functions which are typically disrupted in ASD [57]. Additionally, imaging studies show abnormalities in the limbic region of young subjects [58]. To investigate anatomical abnormalities, we stained P13 brain slices using H&E and Nissl staining. Visible reduction of cells in the hippocampal CA1-subculum area was detected in VPA mice (Fig 2E).

### Dendritic spine density was aberrantly increased in VPA mice

**Fig 3:**
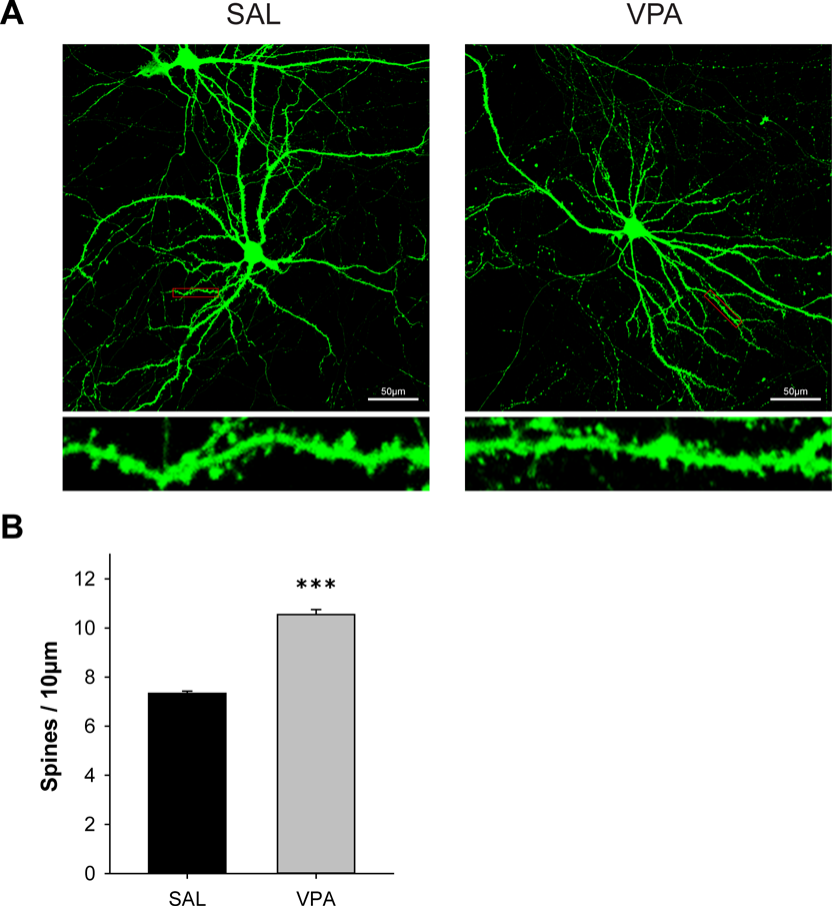
VPA mice showed increased dendritic spine density. Cortical neuron cultures were obtained on E18 from fetuses prenatally exposed to SAL or VPA on E13, and the cells were maintained until DIV 19–20. (*n* = 3 mice for each group, *n* = 3–6 cells for each mouse, 3–6 branches were analyzed per cell.) **(A)** Representative fluorescence images of dendritic spines in primary neuron cultures. **(B)** Quantification of spine density of spine density on secondary dendrites 50–100 μm away from the center of the soma. VPA mice exhibited a significant increase in spine density. (* significantly different among groups, *** *p* < 0.001, *n* = 18 for SAL, *n* = 13 for VPA).

Synaptic regulation is a key mechanism of memory and intelligence. There are several synaptic irregularities in ASD, such as synaptic formation [59], synaptic connection [28, 60], synaptic maintenance and elimination [61]. Studies show that cortical neurons mediate social and emotional communication as well as higher functioning and synaptic abnormalities in these regions may provide insight in understanding ASD [62, 63]. To investigate whether dendritic spines were altered, we used primary cultured cortical neurons from VPA and SAL E18 fetuses. Interestingly, we found an increase in dendritic spine density in VPA primary neurons compared to SAL primary neurons (*t*(29) = -15.44, *p* < 0.001, *n* = 18, 13) (Fig 3). This data suggests synaptic connectivity alternations in VPA similar to those found in other ASD model mice.

### PTEN level was decreased and p-AKT level was increased in VPA mice

**Fig 4:**
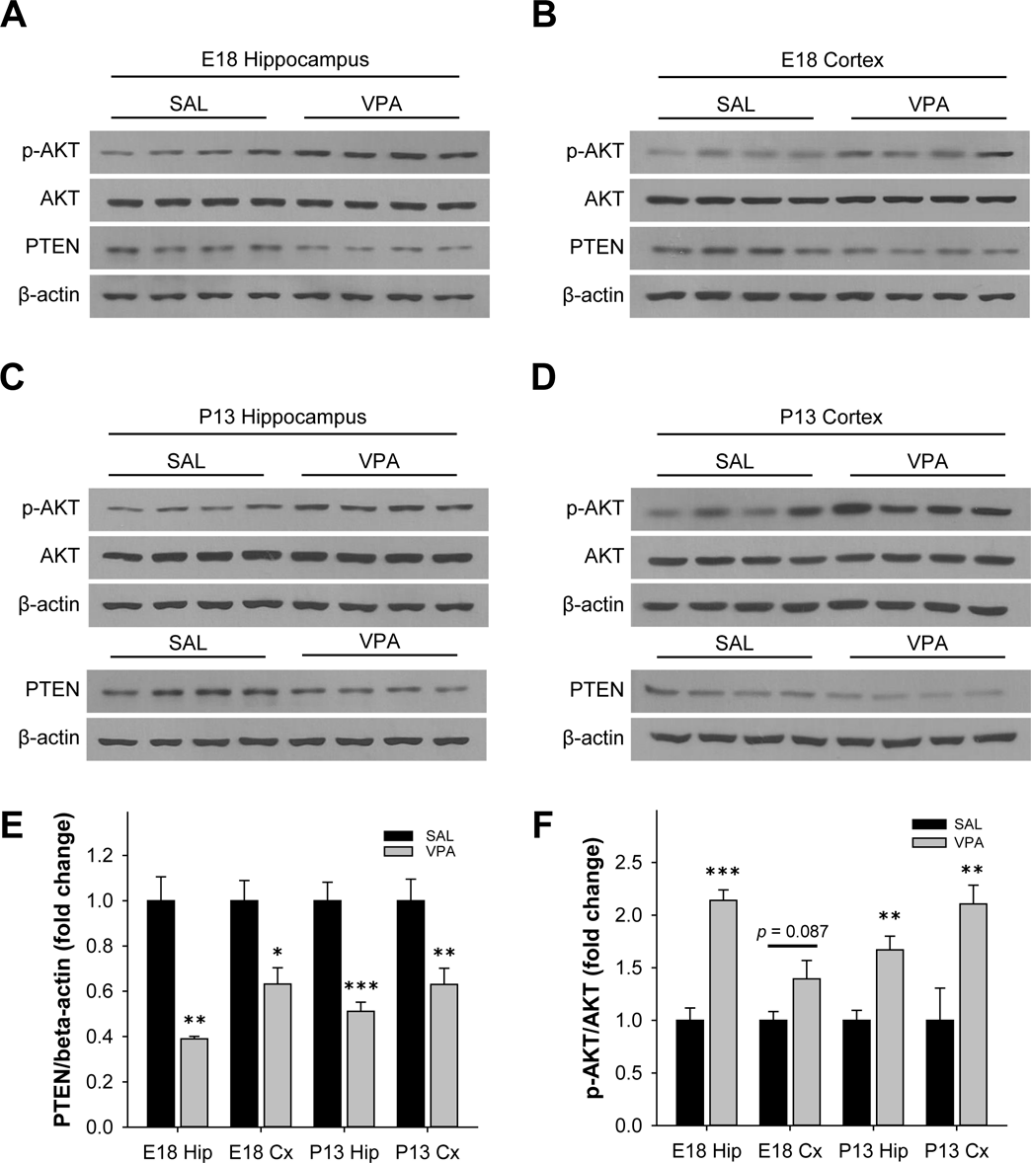
VPA mice showed altered PTEN and p-AKT/AKT ratio in the brain. The protein level of PTEN and p-AKT in the hippocampus and cerebral cortex was examined by western blotting. **(A-D)** Representative blots of p-AKT, AKT, PTEN, and β-actin in the hippocampus and cortex at E18 and P13. **(E-F)** Densitometric analysis of western blots. Phosphorylated-AKT expression was normalized by AKT and PTEN expression was normalized by β-actin. (* significantly different from SAL, * *p* < 0.05, ** *p* < 0.01, *** *p* < 0.001, *n* = 4 for E18 SAL, E18 VPA, and P13 SAL, *n* = 8 for P13 VPA).

Fetuses from E18 SAL and VPA injected mice were compared to determine PTEN levels in the hippocampus and cortex. VPA fetuses showed significant reduction of PTEN in the hippocampus and cortex compared to SAL (E18 hippocampus, *t*(6) = 5.75, *p* < 0.01; E18 cortex, *t*(6) = 3.21, *p* < 0.05; *n* = 4 per group) (Fig 4A, 4B and 4E). In order to determine if PTEN levels were reduced throughout development, PTEN levels on P13 were also investigated. Intriguingly, PTEN expression was also reduced in both the hippocampus and cortex of P13 VPA pups in contrast to SAL (P13 hippocampus, *t*(10) = 6.12, *p* < 0.001; P13 cortex, *t*(10) = 3.01, *p* < 0.05; *n* = 4, 8) (Fig 4C, 4D and 4E).

**Fig 5:**
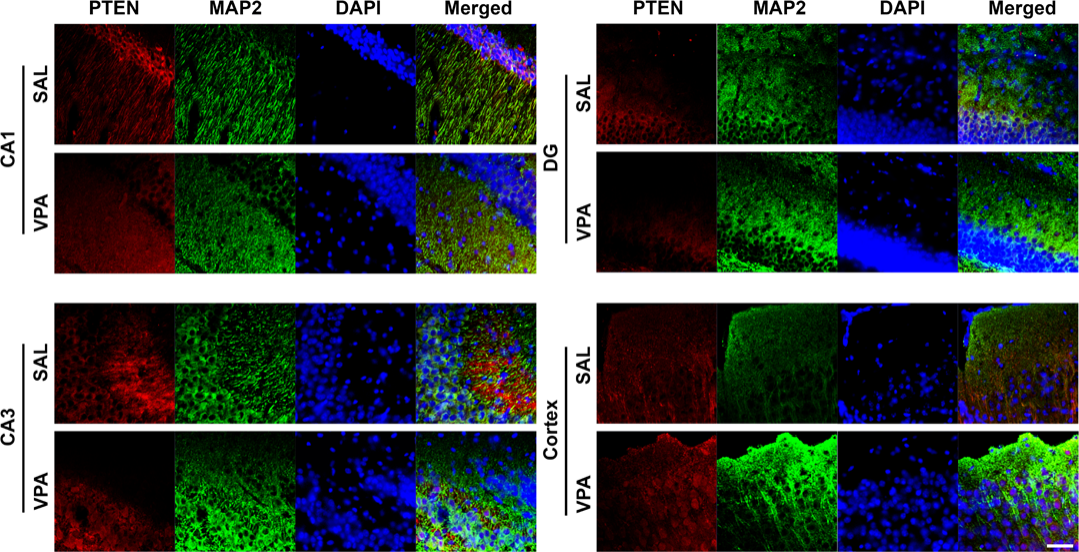
VPA mice showed decreased PTEN expression in multiple brain areas. Representative immunofluorescence images of CA1, CA3, DG of the hippocampus, and cortex stained with PTEN (red), MAP2 (green), DAPI (blue). Scale bar indicates 50 μm.

Since PTEN is a major regulator in the PI3K/AKT/mTOR pathway, we also measured the changes in p-AKT and AKT to find that in E18 VPA hippocampus, the ratio of p-AKT/AKT was significantly increased (E18 hippocampus, *t*(6) = -7.40, *p* < 0.05, *n* = 4 per group) (Fig 4A and 4F). However, the reduction of p-AKT/AKT ratio in the cortex on E18 did not reach significance (E18 cortex, *t*(6) = -2.05, *p* = 0.087, *n* = 4 per group) (Fig 4C and 4F). Furthermore, p-AKT/AKT ratio was significantly increased in both P13 VPA hippocampus and cortex (P13 hippocampus, *t*(10) = -3.35, *p* < 0.01; P13 cortex, *t*(10) = -3.36, *p* < 0.01; *n =* 4, 8) (Fig 4C, 4D and 4F). To determine the localization of expression, immunohistochemistry was performed on P13 SAL and VPA mice and found that PTEN expression was reduced in CA1, CA3, and dentate gyrus (DG) of the hippocampus as well as the cortex in VPA mice (Fig 5).

## Discussion

Several reported cases of VPA exposure during early pregnancy have shown classical signs of autism, minor malformations, and developmental and behavioral delays [22, 48]. Although the VPA-induced autism mouse model does not directly model human ASD, it does enable elucidation of ASD and its relevant biological mechanisms. VPA mice have been shown to have face [25, 42], construct [64] as well as predictive validity [43]. ASD diagnosis is currently based on behavioral criteria, as no biological marker is yet clinically available [65]. In the present study, we focused on the behavioral and molecular alterations in early postnatal phase by utilizing the VPA model rather than a genetic model in effort to study the underlying mechanisms of ASD before manifestation of typical autistic behavioral phenotypes.

This study demonstrates that VPA-exposed pups show developmental delays and social recognition impairments as assessed with eye opening, self-righting and maternal scent preference in P5-16 (Fig 1). Additionally, we provide the first report, to our knowledge, of increased dendritic spine density in primary cultured neurons from VPA mice (Fig 3). Our study also identified that the protein level of PTEN is decreased and p-AKT is increased in the brain of VPA pups, compared to SAL pups (Figs 4 and 5).

A recent study suggested that low birth weight may be a risk factor for ASD [51]. In our study, we found that VPA pups were significantly lower in body weight from pre-birth (E18) to juvenile age (P13) when compared to aged matched SAL pups. However, the brain to body weight ratio was larger in E18 VPA mice, suggesting that VPA exposure results in delayed growth and proportionally larger brains. Our results concurrently reflect delayed intrauterine growth in fetal valproate syndrome [66] as well as macroencephaly observed in PTEN mutations [35].

Eye opening and self-righting reflex are well known developmental milestones in mice. Similar diagnostic tools using motor milestones in development are being used for early detection of autism [67]. Our results show that VPA mice show a significant delay in eye opening in comparison to age-matched pups. Although the exact mechanism of eye opening has yet to be elucidated, the neurodevelopmental delay in VPA pups is similar to previous studies providing construct validity [28]. Self-righting reflex can be described as a three-neuron arc system composed of primary vestibular neurons, vestibular nuclei neurons and target motor-neurons, which simultaneously requires the activation of the cerebellum [68, 69]. The increased latency to self-right observed in the VPA group can be explained by reports that show severe neuronal loss in the somatosensory and motor neurons as well as in the brainstem and cerebellum of VPA treated animals [70, 71].

Social interaction impairment is one of the core symptoms in ASD, and it is imperative to determine social impairment in animal studies. Several studies have employed the use of ultrasonic vocalization or nest seeking test at postnatal developmental periods [72]. Ultrasonic vocalization and nest seeking test both have limitations in neglecting the recognition of familiar and stranger in comparison to the three-chamber social interaction assay. To determine social interaction and recognition we used a modified version of maternal scent preference test. Since social interaction in mice is heavily based on olfactory cues, with the use of familiar bedding and stranger bedding we were able to demonstrate social impairment in earlier dates. In a previous study, the nest seeking response was delayed in VPA pups on P9 but this delay was no longer observed in P13 [26]. In the same context, in the maternal scent preference test performed on P14, both groups did not show difference in total time spent in zones with either scent. However, the VPA group failed to distinguish familiar and stranger scent. These results are similar to those found while conducting three chamber assay with PTEN [37], FMR1 [73], and NLGN3 genetic model mice [74].

Minor neuroanatomical malformations are common in ASD patients as well as fetal valproate syndrome patients. VPA studies have shown a reduction in the number of motor neurons from hypoglossal and oculomotor neuron [71], decreased number of Purkinje cells in the cerebellum [75], reduction in the number of parvalbumin-positive inhibitory neurons in the neocortex [76], and loss of lower layers of the prefrontal cortex and lower somatosensory cortex [77]. In our study, we demonstrate that VPA hippocampus shows hypocellularity in the CA1-subiculum, which is a parallel finding with PTEN conditional knockout mice [38]. Taking together that the long-term potentiation in the CA1-subiculum is diminished by social isolation [78], the anatomical changes observed in VPA mice may be relevant to social behavior deficits.

Here we provide the first report, to our knowledge, that primary cultured neurons from VPA-treated embryos present an increase in dendritic spine density. While preparing primary cultured neurons of BALB/c mice, cultures from VPA-treated group did not survive long enough until spine maturation, hence an alternative strain of mice (C57BL/6) was used for spine counting. Both strains of mice are widely used in research for behavior and molecular changes in the VPA-induced autism model [79]. The changes observed in spines of primary neurons have high significance considering the sparsity of non-genetic *in vitro* material for autism research. FMR1 knockout mice and PTEN conditional knockout mice also exhibit an increase in dendritic spine density [32, 80, 81]. In contrast, MeCP2-deficient mice show to have decreased dendritic spine density [82]. A recent report demonstrated that ASD mouse models show upregulation in the dynamics of PSD-95-positive spines, whereas gephyrin-positive spines were unaffected [83]. Human fMRI studies show conflicting evidence of both hypo- and hyperconnectivity in ASD which vary among regions [84]. These various predictions of connectional changes and its contribution to autistic behavior have not been systematically explored in ASD, and whether the change in spines is due to synaptic maintenance or synaptic elimination is yet to be established.

In summary, VPA-induced autism mice show several similarities to PTEN conditional knockout mice, including proportionally increased brain weight, autistic behavioral symptoms, anatomical changes in the CA1 region of the hippocampus and increased dendritic spines. Perturbations in the two autism models may share a common background, which is the PTEN/AKT pathway. The convergence of this environmentally-induced animal model with a genetic model strongly suggests PTEN as a common molecular target in ASD. Further research is particularly needed to elucidate the molecular mechanism by which the expression of PTEN is downregulated by VPA, and its effect on neural connectivity. The successful elucidation of this mechanism may expand the possibility to adapt PTEN as a major causal gene in the pathogenesis and also utilize it in diagnostics and therapeutics for clinical treatment of ASD.

## Supporting Information

S1 Fig Whole blots of representative data in Fig 4A and 4B. (PDF)

S2 Fig Whole blots of representative data in Fig 4C and 4D. (PDF)

S1 Table Raw data of body weight on P5-13. (PDF)

S2 Table Raw data of self-righting on P5-9. (PDF)

S3 Table Raw data of eye opening on P12-16. (PDF)

S4 Table Raw data maternal scent preference. (PDF)

S5 Table Raw data of body and brain weight on E18. (PDF)

S6 Table Raw data of body and brain weight on P13. (PDF)

S7 Table Raw data of spine density quantification of primary cultures. (PDF)
