# Correction: Early Behavioral Abnormalities and Perinatal Alterations of PTEN/AKT Pathway in Valproic Acid Autism Model Mice

**DOI:** 10.1371/journal.pone.0157202

**Published:** 2016-06-03

**Authors:** Eun-Jeong Yang, Sangzin Ahn, Kihwan Lee, Usman Mahmood, Hye-Sun Kim

The following information is missing from the Funding section: In addition, this work was supported by the Brain Korea 21 PLUS program.
