# Supplementary figures and images for: Early Behavioral Abnormalities and Perinatal Alterations of PTEN/AKT Pathway in Valproic Acid Autism Model Mice
Source: PLoS One. 2016 Apr 12;11(4):e0153298. doi: 10.1371/journal.pone.0153298 (PMC4829151; doi:10.1371/journal.pone.0153298)

**S8 Figure.** Whole blots of representative data in Fig. 4A and 4B.

**E18 Hippocampus**

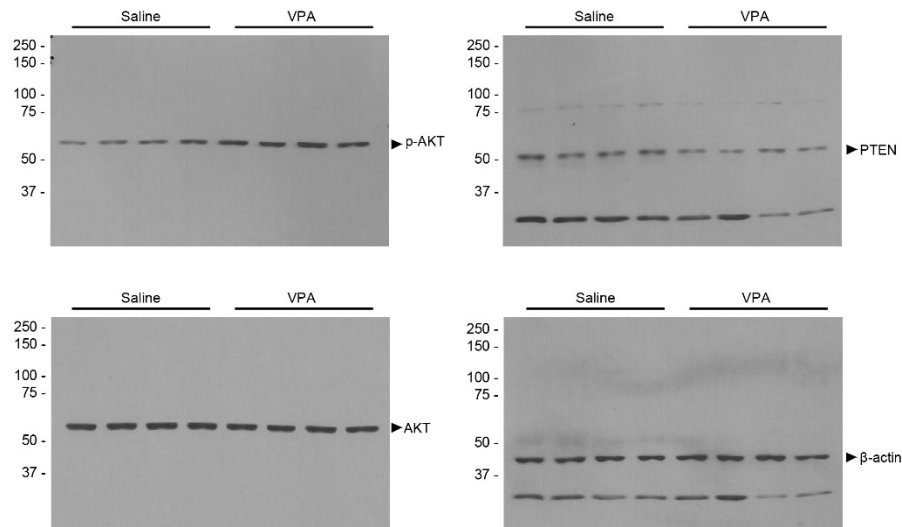

**E18 Cortex**

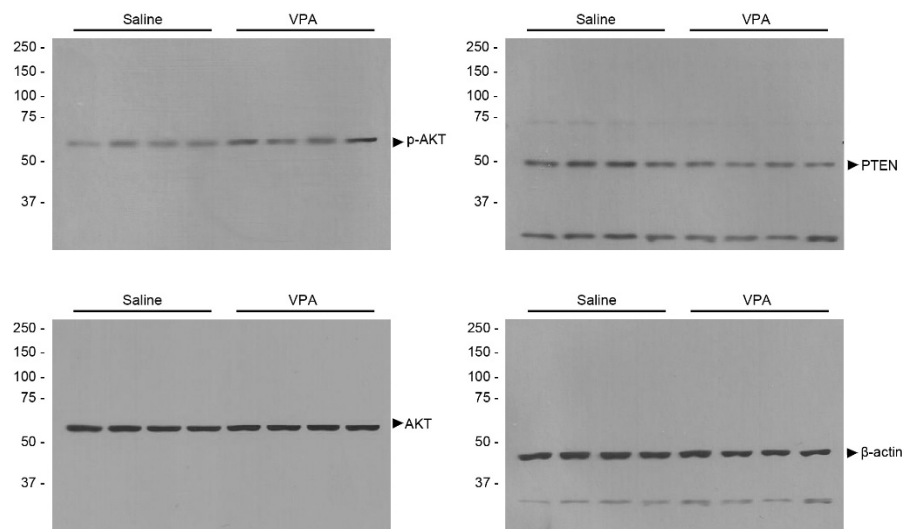

Supplement: S1 Fig — (PDF) [file pone.0153298.s001.pdf]

**S9 Figure.** Whole blots of representative data in Fig. 4C and 4D.

**P13 Hippocampus**

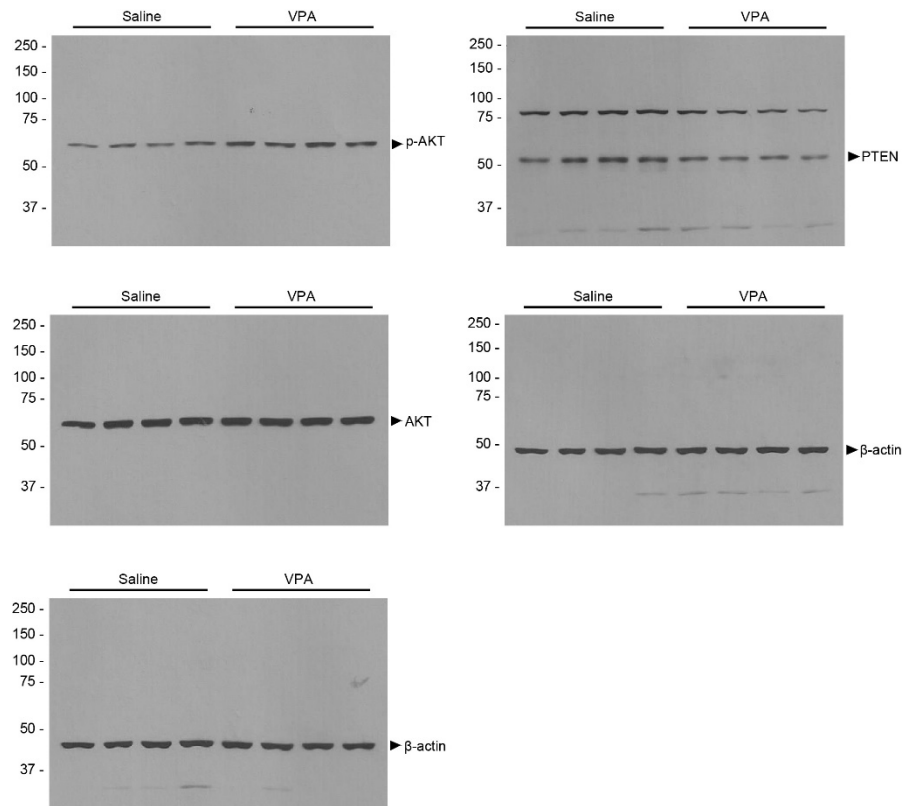

**P13 Cortex**

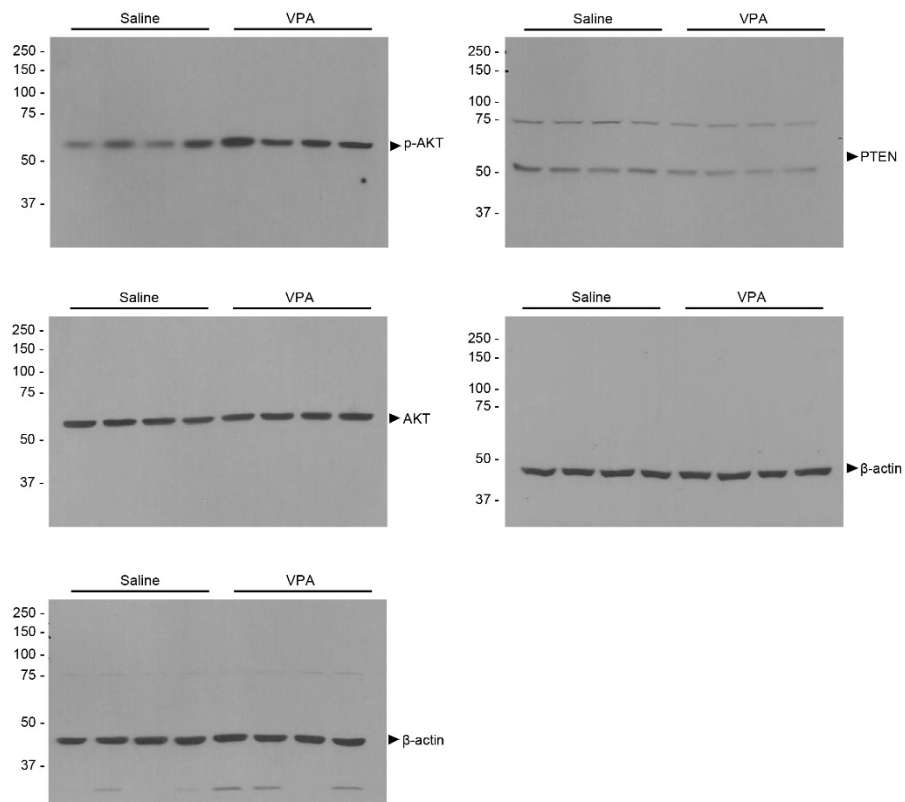

Supplement: S2 Fig — (PDF) [file pone.0153298.s002.pdf]
