# Supplementary material for: Early Behavioral Abnormalities and Perinatal Alterations of PTEN/AKT Pathway in Valproic Acid Autism Model Mice
Source: PLoS One. 2016 Apr 12;11(4):e0153298. doi: 10.1371/journal.pone.0153298 (PMC4829151; doi:10.1371/journal.pone.0153298)
Supplement: S1 Table — (PDF) [file pone.0153298.s003.pdf]

**S1 Table.** Raw data of body weight on P5-13.

**Body weight on P5-13 (g)**

| Group | P5 | P6 | P7 | P8 | P9 | P10 | P11 | P12 | P13 |
|---|---|---|---|---|---|---|---|---|---|
| SAL | 4.3 | 4.7 | 5.3 | 5.7 | 6.5 | 6.9 | 7.5 | 8.1 | 8.3 |
| SAL | 4.5 | 4.9 | 5.2 | 5.6 | 6.1 | 6.5 | 7.1 | 7.7 | 8.1 |
| SAL | 4.3 | 4.9 | 5.2 | 5.9 | 6.3 | 7.0 | 7.5 | 7.9 | 8.4 |
| SAL | 5.0 | 5.6 | 6.5 | 7.3 | 7.8 | 8.4 | 9.0 | 9.2 | 9.6 |
| SAL | 4.9 | 5.8 | 6.7 | 7.5 | 7.8 | 8.4 | 9.0 | 9.5 | 9.9 |
| SAL | 5.1 | 5.8 | 6.5 | 7.3 | 7.5 | 8.3 | 9.0 | 9.3 | 9.6 |
| SAL | 4.8 | 5.6 | 6.5 | 7.3 | 7.6 | 8.2 | 8.9 | 9.4 | 9.7 |
| SAL | 4.9 | 5.7 | 6.7 | 7.3 | 7.4 | 8.3 | 9.1 | 9.6 | 10.0 |
| SAL | 4.0 | 4.7 | 5.4 | 6.0 | 6.5 | 7.0 | 7.4 | 7.5 | 7.8 |
| VPA | 4.2 | 5.0 | 5.6 | 6.4 | 7.0 | 7.6 | 8.2 | 8.7 | 9.2 |
| VPA | 3.9 | 4.5 | 5.5 | 6.2 | 6.9 | 7.6 | 8.3 | 9.1 | 9.3 |
| VPA | 4.3 | 4.9 | 5.7 | 6.3 | 7.1 | 7.6 | 8.1 | 8.6 | 9.0 |
| VPA | 4.7 | 5.2 | 5.9 | 6.8 | 7.4 | 8.0 | 8.5 | 9.3 | 9.6 |
| VPA | 4.5 | 5.2 | 6.0 | 6.4 | 7.3 | 7.9 | 8.4 | 9.1 | 9.4 |
| VPA | 3.8 | 4.0 | 4.8 | 5.2 | 5.7 | 6.1 | 6.4 | 6.8 | 7.1 |
| VPA | 4.2 | 4.9 | 5.3 | 5.9 | 6.5 | 6.8 | 7.0 | 7.4 | 7.5 |
| VPA | 3.6 | 4.0 | 4.6 | 5.0 | 5.4 | 5.9 | 6.3 | 6.7 | 7.0 |
| VPA | 3.6 | 4.2 | 4.8 | 5.3 | 5.7 | 6.1 | 6.5 | 6.9 | 7.0 |
| VPA | 4.0 | 4.6 | 5.1 | 5.6 | 6.2 | 6.2 | 6.1 | 7.1 | 7.5 |
