# Supplementary material for: Early Behavioral Abnormalities and Perinatal Alterations of PTEN/AKT Pathway in Valproic Acid Autism Model Mice
Source: PLoS One. 2016 Apr 12;11(4):e0153298. doi: 10.1371/journal.pone.0153298 (PMC4829151; doi:10.1371/journal.pone.0153298)
Supplement: S2 Table — (PDF) [file pone.0153298.s004.pdf]

**S2 Table.** Raw data of self-righting on P5-9.

**Self-righting on P5-9 (sec)**

| Group | P5 | P6 | P7 | P8 | P9 |
|---|---|---|---|---|---|
| SAL | 25.97 | 4.53 | 1.59 | 0.64 | 1.27 |
| SAL | 25.07 | 14.08 | 3.03 | 1.81 | 0.57 |
| SAL | 27.03 | 21.14 | 3.49 | 1.86 | 0.72 |
| SAL | 16.99 | 15.95 | 4.01 | 1.18 | 1.26 |
| SAL | 17.13 | 19.59 | 19.90 | 1.22 | 0.63 |
| SAL | 17.53 | 14.77 | 3.62 | 1.75 | 0.81 |
| SAL | 15.13 | 8.91 | 1.74 | 1.97 | 1.01 |
| SAL | 14.71 | 17.86 | 3.23 | 6.76 | 0.80 |
| SAL | 18.49 | 6.60 | 10.86 | 11.69 | 1.18 |
| VPA | 23.24 | 20.57 | 15.21 | 12.72 | 5.52 |
| VPA | 8.66 | 12.98 | 9.69 | 3.28 | 2.12 |
| VPA | 21.44 | 22.93 | 13.71 | 13.78 | 2.68 |
| VPA | 6.01 | 13.18 | 5.16 | 9.89 | 2.71 |
| VPA | 20.85 | 6.36 | 21.38 | 3.23 | 2.69 |
| VPA | 30.00 | 22.80 | 6.23 | 30.00 | 14.22 |
| VPA | 18.67 | 15.56 | 21.51 | 19.16 | 2.02 |
| VPA | 23.31 | 4.61 | 11.77 | 1.65 | 2.07 |
| VPA | 21.38 | 30.00 | 23.94 | 9.11 | 11.57 |
| VPA | 30.00 | 22.44 | 2.48 | 3.93 | 8.77 |
