# Supplementary material for: Early Behavioral Abnormalities and Perinatal Alterations of PTEN/AKT Pathway in Valproic Acid Autism Model Mice
Source: PLoS One. 2016 Apr 12;11(4):e0153298. doi: 10.1371/journal.pone.0153298 (PMC4829151; doi:10.1371/journal.pone.0153298)
Supplement: S3 Table — (PDF) [file pone.0153298.s005.pdf]

S3 Table. Raw data of eye opening on P12-16.

Eye opening score on P12-16

Group	P5	P6	P7	P8	P9
SAL	0	1	2	2	2
SAL	0	0	2	2	2
SAL	0	0	1	2	2
SAL	0	0	1	2	2
SAL	0	2	2	2	2
SAL	0	2	2	2	2
SAL	0	1	2	2	2
SAL	0	2	2	2	2
SAL	0	0	2	2	2
VPA	0	0	0	1	1
VPA	0	0	2	2	2
VPA	0	0	1	2	2
VPA	0	0	1	2	2
VPA	0	0	2	2	2
VPA	0	0	2	2	2
VPA	0	0	1	2	2
VPA	0	0	1	2	2
VPA	0	0	1	2	2
VPA	0	1	2	2	2
