# Supplementary material for: Early Behavioral Abnormalities and Perinatal Alterations of PTEN/AKT Pathway in Valproic Acid Autism Model Mice
Source: PLoS One. 2016 Apr 12;11(4):e0153298. doi: 10.1371/journal.pone.0153298 (PMC4829151; doi:10.1371/journal.pone.0153298)
Supplement: S4 Table — (PDF) [file pone.0153298.s006.pdf]

S4 Table. Raw data of maternal scent preference.

Maternal scent preference (sec)

Group	Familiar	Center	Stranger
SAL	24.71	35.59	0.00
SAL	38.68	22.10	0.00
SAL	31.03	12.73	16.55
SAL	36.69	10.59	15.15
SAL	32.46	23.99	4.95
SAL	21.90	38.28	0.00
SAL	13.47	46.75	0.00
SAL	47.04	12.77	0.00
SAL	11.15	41.01	7.67
VPA	51.61	8.65	0.00
VPA	52.70	8.47	0.00
VPA	16.84	9.77	34.12
VPA	23.53	8.86	29.28
VPA	0.00	20.35	37.01
VPA	6.35	48.82	5.13
VPA	30.73	13.63	16.52
VPA	7.63	41.46	10.64
VPA	0.00	8.27	44.99
VPA	7.70	10.13	26.80
