# Supplementary material for: Early Behavioral Abnormalities and Perinatal Alterations of PTEN/AKT Pathway in Valproic Acid Autism Model Mice
Source: PLoS One. 2016 Apr 12;11(4):e0153298. doi: 10.1371/journal.pone.0153298 (PMC4829151; doi:10.1371/journal.pone.0153298)
Supplement: S5 Table — (PDF) [file pone.0153298.s007.pdf]

S5 Table. Raw data of body and brain weight on E18.

Body and brain weight on E18 (μg)

Group	Body	Brain	Body/brain ratio
SAL	1338.2	75.3	0.0563
SAL	1249.0	73.6	0.0589
SAL	1313.6	77.7	0.0592
SAL	1124.2	74.7	0.0664
SAL	1205.3	68.5	0.0568
SAL	1260.9	71.4	0.0566
SAL	1282.0	67.4	0.0526
SAL	1321.6	66.6	0.0504
SAL	1273.8	70.6	0.0554
SAL	1238.5	72.3	0.0584
VPA	926.7	68.2	0.0736
VPA	741.5	55.7	0.0751
VPA	837.3	56.5	0.0675
VPA	823.4	58.3	0.0708
VPA	986.6	68.0	0.0689
VPA	793.5	50.7	0.0639
VPA	851.8	66.6	0.0782
VPA	1056.4	68.2	0.0646
VPA	963.0	53.8	0.0559
