# Supplementary material for: Early Behavioral Abnormalities and Perinatal Alterations of PTEN/AKT Pathway in Valproic Acid Autism Model Mice
Source: PLoS One. 2016 Apr 12;11(4):e0153298. doi: 10.1371/journal.pone.0153298 (PMC4829151; doi:10.1371/journal.pone.0153298)
Supplement: S6 Table — (PDF) [file pone.0153298.s008.pdf]

S6 Table. Raw data of body and brain weight on P13.

Body and brain weight on P13 (μg)

Group	Body	Brain	Body/brain ratio
SAL	9600	405.6	0.0423
SAL	9900	377.3	0.0381
SAL	9600	375.0	0.0391
SAL	10000	397.1	0.0397
SAL	7800	388.6	0.0498
SAL	9200	346.4	0.0377
SAL	9300	334.3	0.0359
SAL	9000	357.6	0.0397
SAL	9600	328.0	0.0342
SAL	9400	344.6	0.0367
VPA	7100	326.4	0.0460
VPA	7500	338.8	0.0452
VPA	7000	310.1	0.0443
VPA	7000	326.8	0.0467
VPA	7000	309.9	0.0443
VPA	9600	405.6	0.0423
VPA	9900	377.3	0.0381
VPA	9600	375.0	0.0391
VPA	10000	397.1	0.0397
