# Supplementary material for: Early Behavioral Abnormalities and Perinatal Alterations of PTEN/AKT Pathway in Valproic Acid Autism Model Mice
Source: PLoS One. 2016 Apr 12;11(4):e0153298. doi: 10.1371/journal.pone.0153298 (PMC4829151; doi:10.1371/journal.pone.0153298)
Supplement: S7 Table — (PDF) [file pone.0153298.s009.pdf]

S7 Table. Raw data of spine density quantification of primary cultures.

Spine density (spines/10μm)

Group	Spines/10μm	Group	Spines/10μm
SAL	7.70	VPA	10.17
SAL	6.75	VPA	9.75
SAL	6.88	VPA	10.50
SAL	7.93	VPA	10.58
SAL	7.07	VPA	9.50
SAL	7.00	VPA	11.21
SAL	7.50	VPA	10.90
SAL	7.50	VPA	10.50
SAL	6.67	VPA	10.80
SAL	7.70	VPA	9.13
SAL	7.58	VPA	11.67
SAL	7.63	VPA	11.21
SAL	7.75	VPA	11.19
SAL	6.92		
SAL	7.83		
SAL	6.90		
SAL	7.50		
SAL	7.20		
